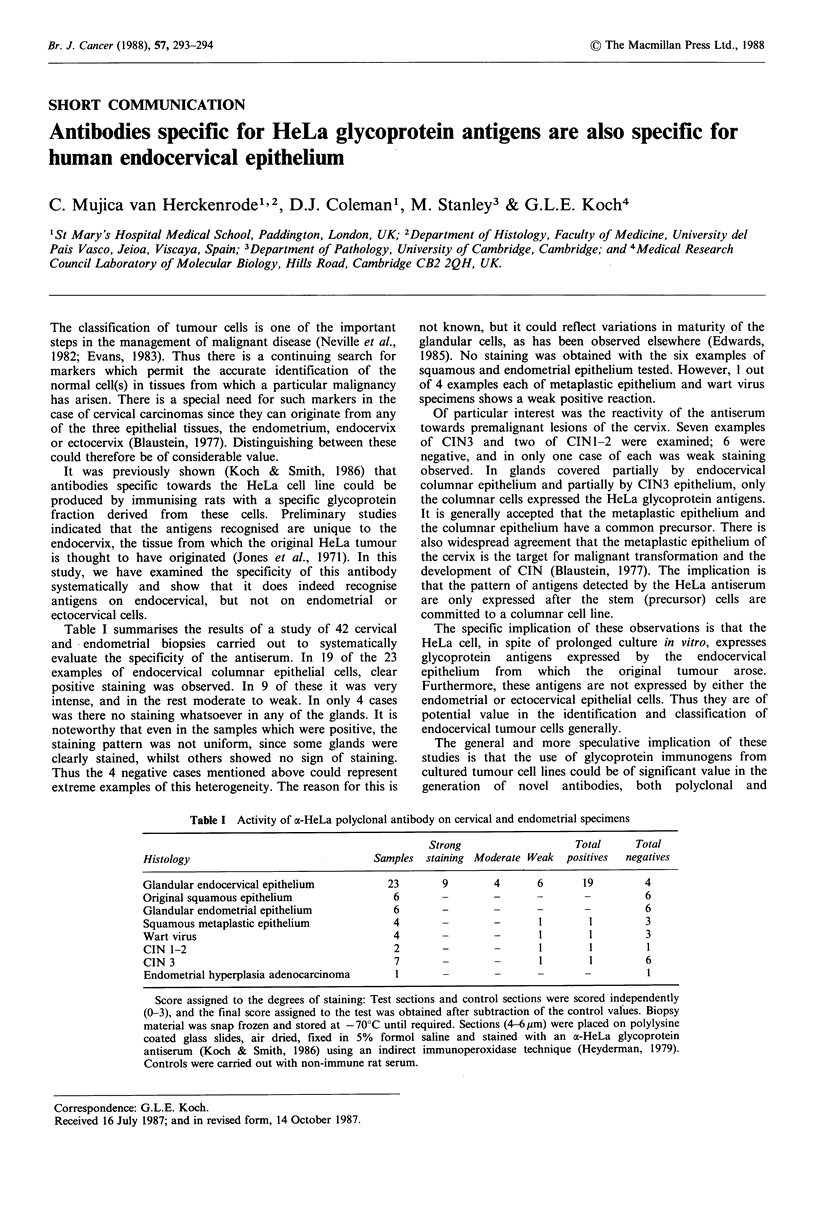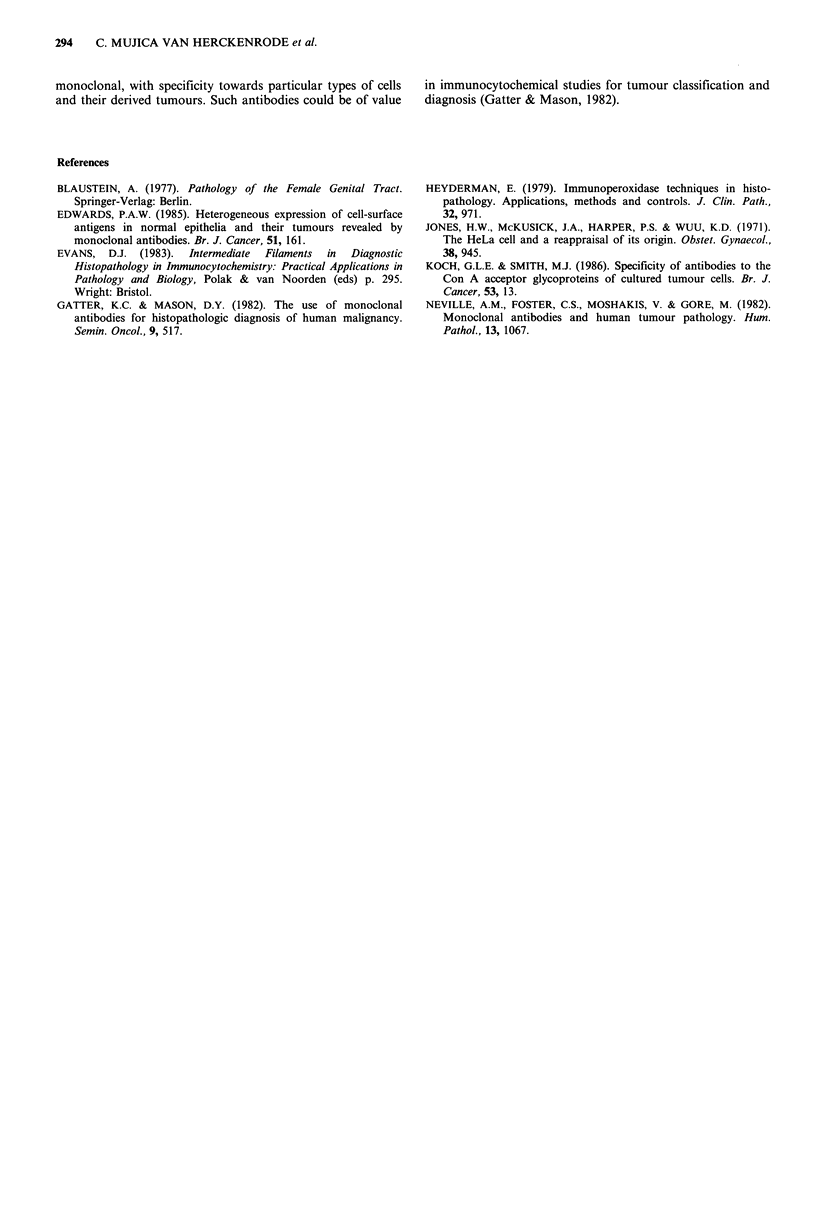# Antibodies specific for HeLa glycoprotein antigens are also specific for human endocervical epithelium.

**DOI:** 10.1038/bjc.1988.64

**Published:** 1988-03

**Authors:** C. Mujica van Herckenrode, D. J. Coleman, M. Stanley, G. L. Koch

**Affiliations:** St Mary's Hospital Medical School, Paddington, London, UK.


*Br. J. Cancer* (1988), **57**, 293–294 © The Macmillan Press Ltd., 1988

SHORT COMMUNICATION

### Antibodies specific for HeLa glycoprotein antigens are also specific for human endocervical epithelium

C. Mujica van Herckenrode[1,2], D.J. Coleman[1], M. Stanley[3] & G.L.E. Koch[4]

[1]*St Mary's Hospital Medical School, Paddington, London, UK;* [2]*Department of Histology, Faculty of Medicine, University del Pais Vasco, Jeioa, Viscaya, Spain;* [3]*Department of Pathology, University of Cambridge, Cambridge; and* [4]*Medical Research Council Laboratory of Molecular Biology, Hills Road, Cambridge CB2 2QH, UK.*

The classification of tumour cells is one of the important steps in the management of malignant disease (Neville *et al.*, 1982; Evans, 1983). Thus there is a continuing search for markers which permit the accurate identification of the normal cell(s) in tissues from which a particular malignancy has arisen. There is a special need for such markers in the case of cervical carcinomas since they can originate from any of the three epithelial tissues, the endometrium, endocervix or ectocervix (Blaustein, 1977). Distinguishing between these could therefore be of considerable value.

It was previously shown (Koch & Smith, 1986) that antibodies specific towards the HeLa cell line could be produced by immunising rats with a specific glycoprotein fraction derived from these cells. Preliminary studies indicated that the antigens recognised are unique to the endocervix, the tissue from which the original HeLa tumour is thought to have originated (Jones *et al.*, 1971). In this study, we have examined the specificity of this antibody systematically and show that it does indeed recognise antigens on endocervical, but not on endometrial or ectocervical cells.

Table I summarises the results of a study of 42 cervical and endometrial biopsies carried out to systematically evaluate the specificity of the antiserum. In 19 of the 23 examples of endocervical columnar epithelial cells, clear positive staining was observed. In 9 of these it was very intense, and in the rest moderate to weak. In only 4 cases was there no staining whatsoever in any of the glands. It is noteworthy that even in the samples which were positive, the staining pattern was not uniform, since some glands were clearly stained, whilst others showed no sign of staining. Thus the 4 negative cases mentioned above could represent extreme examples of this heterogeneity. The reason for this is not known, but it could reflect variations in maturity of the glandular cells, as has been observed elsewhere (Edwards, 1985). No staining was obtained with the six examples of squamous and endometrial epithelium tested. However, 1 out of 4 examples each of metaplastic epithelium and wart virus specimens shows a weak positive reaction.

Of particular interest was the reactivity of the antiserum towards premalignant lesions of the cervix. Seven examples of CIN3 and two of CIN1–2 were examined; 6 were negative, and in only one case of each was weak staining observed. In glands covered partially by endocervical columnar epithelium and partially by CIN3 epithelium, only the columnar cells expressed the HeLa glycoprotein antigens. It is generally accepted that the metaplastic epithelium and the columnar epithelium have a common precursor. There is also widespread agreement that the metaplastic epithelium of the cervix is the target for malignant transformation and the development of CIN (Blaustein, 1977). The implication is that the pattern of antigens detected by the HeLa antiserum are only expressed after the stem (precursor) cells are committed to a columnar cell line.

The specific implication of these observations is that the HeLa cell, in spite of prolonged culture *in vitro*, expresses glycoprotein antigens expressed by the endocervical epithelium from which the original tumour arose. Furthermore, these antigens are not expressed by either the endometrial or ectocervical epithelial cells. Thus they are of potential value in the identification and classification of endocervical tumour cells generally.

The general and more speculative implication of these studies is that the use of glycoprotein immunogens from cultured tumour cell lines could be of significant value in the generation of novel antibodies, both polyclonal and

**Table I** Activity of α-HeLa polyclonal antibody on cervical and endometrial specimens

| *Histology* | *Samples* | *Strong staining* | *Moderate* | *Weak* | *Total positives* | *Total negatives* |
|---|---|---|---|---|---|---|
| Glandular endocervical epithelium | 23 | 9 | 4 | 6 | 19 | 4 |
| Original squamous epithelium | 6 | – | – | – | – | 6 |
| Glandular endometrial epithelium | 6 | – | – | – | – | 6 |
| Squamous metaplastic epithelium | 4 | – | – | 1 | 1 | 3 |
| Wart virus | 4 | – | – | 1 | 1 | 3 |
| CIN 1–2 | 2 | – | – | 1 | 1 | 1 |
| CIN 3 | 7 | – | – | 1 | 1 | 6 |
| Endometrial hyperplasia adenocarcinoma | 1 | – | – | – | – | 1 |

Score assigned to the degrees of staining: Test sections and control sections were scored independently (0–3), and the final score assigned to the test was obtained after subtraction of the control values. Biopsy material was snap frozen and stored at −70°C until required. Sections (4–6 μm) were placed on polylysine coated glass slides, air dried, fixed in 5% formol saline and stained with an α-HeLa glycoprotein antiserum (Koch & Smith, 1986) using an indirect immunoperoxidase technique (Heyderman, 1979). Controls were carried out with non-immune rat serum.

Correspondence: G.L.E. Koch.
Received 16 July 1987; and in revised form, 14 October 1987.

monoclonal, with specificity towards particular types of cells and their derived tumours. Such antibodies could be of value in immunocytochemical studies for tumour classification and diagnosis (Gatter & Mason, 1982).

### References

BLAUSTEIN, A. (1977). *Pathology of the Female Genital Tract*. Springer-Verlag: Berlin.

EDWARDS, P.A.W. (1985). Heterogeneous expression of cell-surface antigens in normal epithelia and their tumours revealed by monoclonal antibodies. *Br. J. Cancer*, **51**, 161.

EVANS, D.J. (1983). *Intermediate Filaments in Diagnostic Histopathology in Immunocytochemistry: Practical Applications in Pathology and Biology*, Polak & van Noorden (eds) p. 295. Wright: Bristol.

GATTER, K.C. & MASON, D.Y. (1982). The use of monoclonal antibodies for histopathologic diagnosis of human malignancy. *Semin. Oncol.*, **9**, 517.

HEYDERMAN, E. (1979). Immunoperoxidase techniques in histopathology. Applications, methods and controls. *J. Clin. Path.*, **32**, 971.

JONES, H.W., MCKUSICK, J.A., HARPER, P.S. & WUU, K.D. (1971). The HeLa cell and a reappraisal of its origin. *Obstet. Gynaecol.*, **38**, 945.

KOCH, G.L.E. & SMITH, M.J. (1986). Specificity of antibodies to the Con A acceptor glycoproteins of cultured tumour cells. *Br. J. Cancer*, **53**, 13.

NEVILLE, A.M., FOSTER, C.S., MOSHAKIS, V. & GORE, M. (1982). Monoclonal antibodies and human tumour pathology. *Hum. Pathol.*, **13**, 1067.